# Supplementary material for: Sustained decline in tobacco purchasing in Denmark during the COVID-19 pandemic
Source: Commun Med (Lond). 2022 Aug 2;2:96. doi: 10.1038/s43856-022-00160-1 (PMC9344799; doi:10.1038/s43856-022-00160-1)
Supplement: Supplementary file 1 — Supplementary Information [file 43856_2022_160_MOESM1_ESM.pdf]

Supplementary Materials

Supplementary Figure S1. Coefficients Plot of Weekly Proportion of Cigarette Purchases and Quantity by Demographic Group.

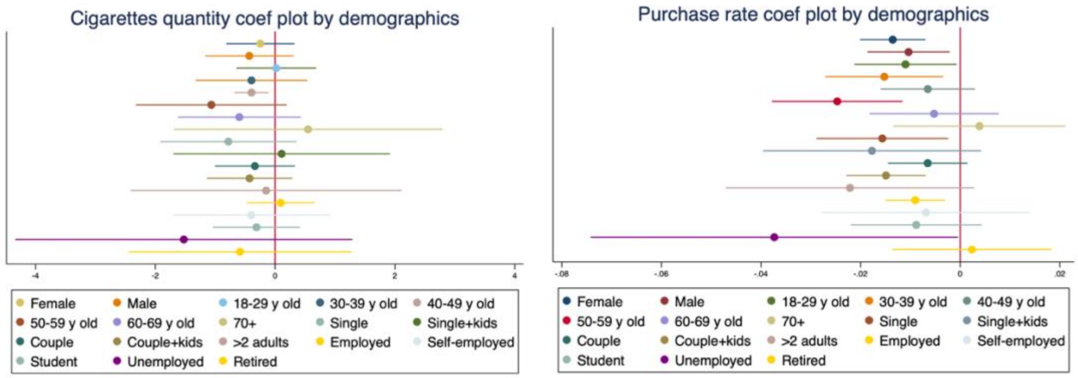

Note: Error bars shows the 95% confidence interval. N=4042.

Supplementary Figure S2. Convenience store and grocery store sales 2019-2020 (Index: 2015=100)

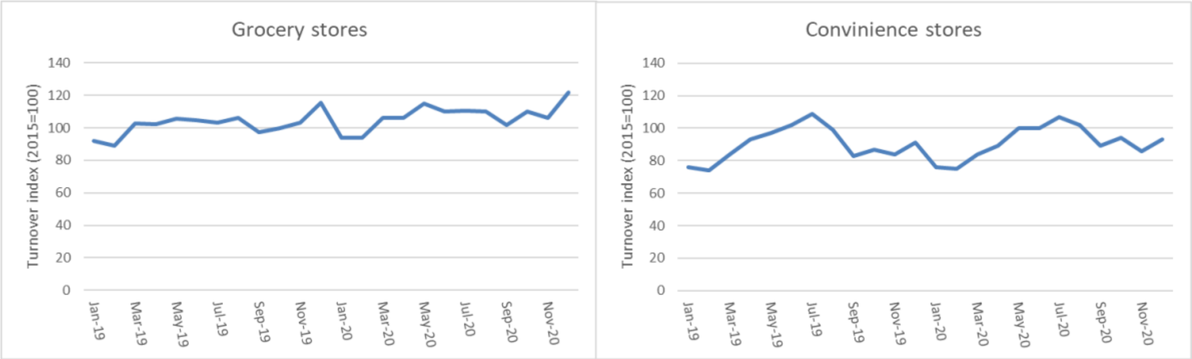

Note: The total amount of monthly sales in convenience stores and grocery stores (in Danish: "Købmand og døgnkiosker" & "Detailomsætningsindex"), indexed to average monthly sales in 2015. Data source: Statistics Denmark, www.statistikbanken.dk/DETA151 + www.statistikbanken.dk/DETA152.

Supplementary Figure S3. National Cigarette and Alcohol Sales by Year.

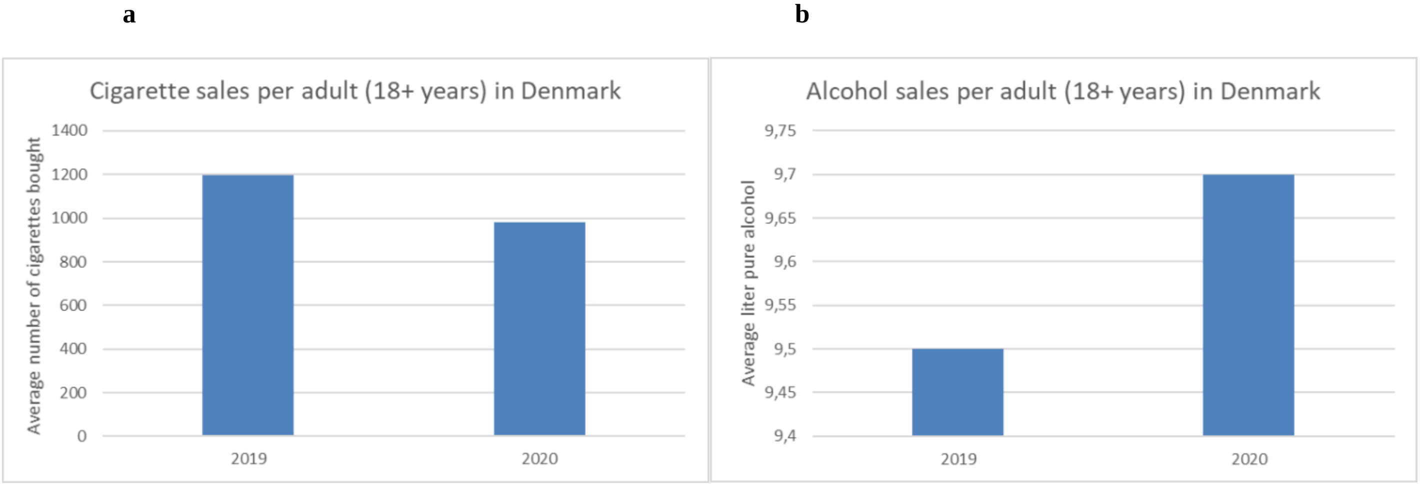

Note: **a** shows the number of cigarettes that an average Danish adult bought in 2019 and 2020; **b** shows the liter of pure alcohol an average Danish adult bought in 2019 and 2020. The pure alcohol measure is determined by calculating the alcohol share (in liters) of each alcohol-containing product sold. Data source: Statistics Denmark: www.statistikbanken.dk/ALKO2

Supplementary Figure S4. Weekly Average Prices of Cigarettes Observed in our Dataset.

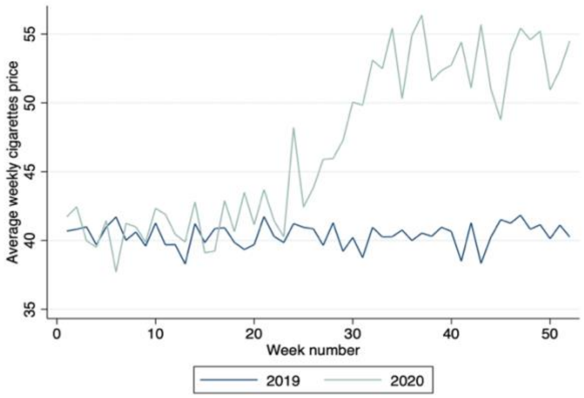

Supplementary Figure S5. Time Trend of Google Searches for Keywords "Corona and Smoking".

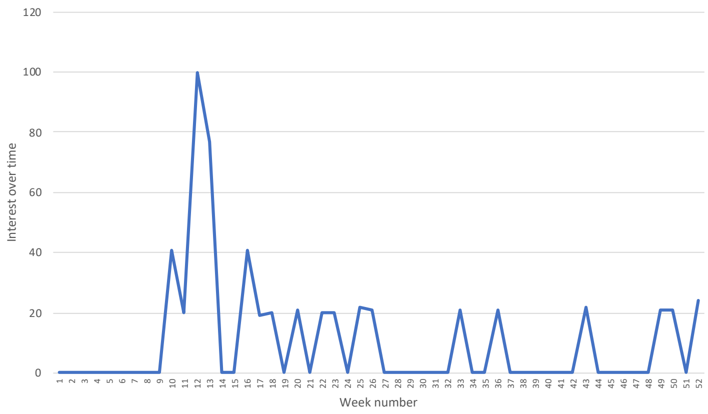

Note: the numbers represent the "search interest" relative to the highest point on the chart for the given region and time (Denmark, 2020). A value of 100 is the peak popularity for the term; while a value of 50 corresponds to half as popular, and 0 is included if there is not enough data to be reported for the term (Google Trends).

Supplementary Table S1. Demographics in a; Smoking Habit Baselines in b.

a

	National Population	Sample
Sample size (N)	--	4042
Demographics		
Female	50.3%	65%
Age		
20-29	13%	15%
30-39	12%	27.3%
40-49	13%	24.3%
50-59	14%	18.4%
60-69	11%	11.8%
70-79	10%	3.2%
Employed	66%	72%
Household Income ($)		
<47.4k	34%	19.5%
48-79k	26%	22.5%
>80k	39%	58%
Household Type		
Single	24%	16.7%
Single + children	4%	8.5%
Couple	33%	29.6%
Couple + children	24%	39.5%
3 or more adults	15%	5.7%
Capital Region (Copenhagen)	23%	42%

b

Baselines	All	Non-smokers	Smokers	Occasionals	Regulars
Individuals N	4042	3059	983	658	325
Cigarettes purchase rate	0.0491	0	0.2021	0.0576	0.4946
p-value			0.0000		0.0000
Cigarettes quantity	3.3376	0	13.7240	1.16816	38.1053
p-value			0.0000		0.0000
Alcohol purchase rate	0.2030	0.1897	0.2445	0.2340	0.2657
p-value			0.0000		0.0865
Alcohol quantity	0.7936	0.7304	0.9905	0.8876	1.1988
p-value			0.0000		0.2561

Note: p-values from rank-sum tests.

Supplementary Table S2. Sensitivity Check with Purchase Rate of all Types of Tobacco.

	Any kind of tobacco									
	**All**	**All+price**	**Non-smokers**	**Non-smokers+price**	**Smokers**	**Smokers+price**	**Occasionals**	**Occasionals+price**	**Regulars**	**Regulars+price**
Spring	0.002	0.001	-0.000	-0.000	0.009*	0.005	0.008*	0.007	0.013	0.000
	(0.001)	(0.001)	(0.000)	(0.000)	(0.005)	(0.006)	(0.004)	(0.005)	(0.014)	(0.014)
Summer	-0.001	-0.000	-0.000	-0.000	-0.003	-0.001	0.006	0.007	-0.023*	-0.017
	(0.001)	(0.001)	(0.000)	(0.000)	(0.005)	(0.005)	(0.005)	(0.005)	(0.014)	(0.014)
Fall	-0.004***	-0.003*	-0.000	-0.000	-0.014***	-0.010*	0.001	0.002	-0.044***	-0.032**
	(0.001)	(0.001)	(0.000)	(0.000)	(0.005)	(0.005)	(0.005)	(0.005)	(0.013)	(0.014)
COVID-19	0.006***	0.006***	0.000	0.000	0.026***	0.025***	0.000	-0.000	0.077***	0.074***
	(0.002)	(0.002)	(0.000)	(0.000)	(0.007)	(0.007)	(0.006)	(0.005)	(0.018)	(0.018)
Year (2020)	0.003	0.003	0.003***	0.003***	0.003	0.003	0.003	0.003	0.004	0.004
	(0.002)	(0.002)	(0.000)	(0.000)	(0.008)	(0.008)	(0.006)	(0.006)	(0.022)	(0.022)
COVID-19 X Year	-0.016***	-0.012***	0.001	0.001	-0.066***	-0.053***	-0.008	-0.005	-0.183***	-0.149***
	(0.002)	(0.002)	(0.000)	(0.001)	(0.009)	(0.008)	(0.006)	(0.006)	(0.023)	(0.021)
Weekly cig price		-0.019***		0.000		-0.077***		-0.016		-0.200***
		(0.006)		(0.002)		(0.023)		(0.018)		(0.057)
Constant	0.045***	0.113***	0.000	-0.001	0.183***	0.469***	0.054***	0.114*	0.446***	1.187***
	(0.001)	(0.021)	(0.000)	(0.007)	(0.006)	(0.082)	(0.004)	(0.066)	(0.015)	(0.208)
N	420368	420368	318136	318136	102232	102232	68432	68432	33800	33800
Degrees of freedom	4041	4041	3058	3058	982	982	657	657	324	324
Clusters (individuals)	4042	4042	3059	3059	983	983	658	658	325	325

* p<0.1, ** p<0.05, *** p<0.01

Supplementary Table S3. Regression on the impact of COVID-19 on cigarette quantity using Inverse Probability Weighting of the sample for the national shares of smokers and non-smokers.

	IPW on cigarette quantity by smoker category									
	**All**	**All+price**	**Non-smokers**	**Non-smokers+price**	**Smokers**	**Smokers+price**	**Occasionals**	**Occasionals+price**	**Regulars**	**Regulars+price**
Spring	0.397***	0.193	0.002	-0.006	1.624***	0.812	0.333	0.225	4.237***	2.001
	(0.134)	(0.138)	(0.032)	(0.027)	(0.543)	(0.560)	(0.205)	(0.208)	(1.579)	(1.640)
Summer	-0.012	0.084	-0.034	-0.030	0.055	0.439	0.077	0.128	0.012	1.068
	(0.135)	(0.131)	(0.027)	(0.027)	(0.547)	(0.533)	(0.236)	(0.228)	(1.587)	(1.544)
Fall	-0.239*	-0.050	-0.029	-0.021	-0.892*	-0.142	0.010	0.110	-2.720*	-0.652
	(0.132)	(0.129)	(0.026)	(0.027)	(0.537)	(0.524)	(0.240)	(0.220)	(1.547)	(1.522)
Week 11 - 52	0.630***	0.587***	0.018	0.017	2.535***	2.362***	-0.114	-0.137	7.899***	7.423***
	(0.168)	(0.165)	(0.024)	(0.025)	(0.684)	(0.669)	(0.256)	(0.253)	(1.971)	(1.930)
Year (2020)	0.215	0.221	0.097***	0.098***	0.582	0.605	0.176	0.179	1.403	1.467
	(0.175)	(0.176)	(0.019)	(0.019)	(0.719)	(0.721)	(0.253)	(0.253)	(2.114)	(2.122)
COVID-19 period ^	-0.949***	-0.400**	0.059**	0.081**	-4.083***	-1.897**	0.542*	0.834***	-13.447***	-7.424***
	(0.215)	(0.188)	(0.025)	(0.037)	(0.875)	(0.764)	(0.290)	(0.297)	(2.503)	(2.203)
Cigarettes price		-3.213***		-0.131		-12.802***		-1.710		-35.260***
		(0.679)		(0.138)		(2.736)		(1.079)		(7.845)
Constant	2.784***	14.686***	0.000	0.486	11.449***	58.874***	1.663***	7.998**	31.262***	161.879***
	(0.221)	(2.532)	(.)	(0.512)	(0.851)	(10.164)	(0.143)	(3.990)	(2.180)	(28.859)
N	420368	420368	318136	318136	102232	102232	68432	68432	33800	33800
Degrees of freedom	4041	4041	3058	3058	982	982	657	657	324	324
Clusters (individuals)	4042	4042	3059	3059	983	983	658	658	325	325

* p<0.1, ** p<0.05, *** p<0.01

^ *COVID-19 period* is the coefficient for the interaction of Week 11-52 x Year (2020)

Supplementary Table S4. Full Regression Table on Alcohol Consumption.

a

	Alcohol purchase rate									
	**All**	**All+price**	**Non-smokers**	**Non-smokers+price**	**Smokers**	**Smokers+price**	**Occasionals**	**Occasionals+price**	**Regulars**	**Regulars+price**
Spring	-0.007**	-0.012***	-0.006*	-0.011***	-0.009	-0.015**	-0.006	-0.013	-0.017	-0.019*
	(0.003)	(0.003)	(0.004)	(0.004)	(0.007)	(0.007)	(0.008)	(0.008)	(0.012)	(0.012)
Summer	-0.022***	-0.020***	-0.019***	-0.017***	-0.031***	-0.028***	-0.027***	-0.023***	-0.039***	-0.038***
	(0.003)	(0.003)	(0.004)	(0.004)	(0.007)	(0.007)	(0.008)	(0.008)	(0.012)	(0.012)
Fall	-0.013***	-0.008**	-0.010***	-0.006	-0.021***	-0.016**	-0.021**	-0.013	-0.023*	-0.021*
	(0.003)	(0.003)	(0.004)	(0.004)	(0.007)	(0.007)	(0.008)	(0.008)	(0.012)	(0.012)
COVID-19	0.066***	0.065***	0.060***	0.059***	0.082***	0.081***	0.077***	0.075***	0.093***	0.093***
	(0.004)	(0.004)	(0.004)	(0.004)	(0.008)	(0.008)	(0.009)	(0.009)	(0.014)	(0.014)
Year (2020)	0.023***	0.023***	0.023***	0.023***	0.021***	0.022***	0.023***	0.023***	0.019	0.019
	(0.003)	(0.003)	(0.004)	(0.004)	(0.007)	(0.007)	(0.008)	(0.008)	(0.012)	(0.012)
COVID-19 X Year	0.001	0.015***	0.002	0.015***	-0.001	0.015*	0.004	0.025**	-0.012	-0.005
	(0.004)	(0.004)	(0.004)	(0.004)	(0.008)	(0.008)	(0.010)	(0.010)	(0.013)	(0.013)
Cigarettes price		-0.078***		-0.072***		-0.096***		-0.121***		-0.045
		(0.012)		(0.013)		(0.025)		(0.030)		(0.044)
Constant	0.161***	0.449***	0.150***	0.416***	0.194***	0.549***	0.186***	0.634***	0.210***	0.377**
	(0.002)	(0.043)	(0.002)	(0.048)	(0.005)	(0.092)	(0.006)	(0.112)	(0.009)	(0.163)
Baseline 2019	0.2030	0.2030	0.1897	0.1897	0.2445	0.2445	0.2340	0.2340	0.2657	0.2657
N	420368	420368	318136	318136	102232	102232	68432	68432	33800	33800
Degrees of freedom	4041	4041	3058	3058	982	982	657	657	324	324
Clusters (individuals)	4042	4042	3059	3059	983	983	658	658	325	325

* p<0.1, ** p<0.05, *** p<0.01

b

	Alcohol quantity									
	**All**	**All+price**	**Non-smokers**	**Non-smokers+price**	**Smokers**	**Smokers+price**	**Occasionals**	**Occasionals+price**	**Regulars**	**Regulars+price**
Spring	-0.020	-0.046*	0.009	-0.018	-0.110*	-0.132**	-0.109*	-0.127*	-0.112	-0.142
	(0.025)	(0.025)	(0.027)	(0.027)	(0.057)	(0.059)	(0.065)	(0.067)	(0.111)	(0.115)
Summer	-0.102***	-0.090***	-0.067**	-0.054*	-0.212***	-0.202***	-0.153**	-0.144**	-0.334**	-0.320**
	(0.026)	(0.026)	(0.027)	(0.028)	(0.065)	(0.064)	(0.072)	(0.072)	(0.131)	(0.127)
Fall	-0.044*	-0.021	-0.009	0.016	-0.155**	-0.134**	-0.147**	-0.129*	-0.171	-0.143
	(0.026)	(0.028)	(0.027)	(0.031)	(0.063)	(0.062)	(0.069)	(0.069)	(0.131)	(0.126)
COVID-19	0.335***	0.330***	0.289***	0.283***	0.479***	0.474***	0.439***	0.435***	0.559***	0.552***
	(0.026)	(0.026)	(0.027)	(0.027)	(0.064)	(0.064)	(0.074)	(0.074)	(0.121)	(0.121)
Year (2020)	0.098***	0.099***	0.080***	0.081***	0.154***	0.155***	0.086*	0.087*	0.291**	0.292**
	(0.022)	(0.022)	(0.023)	(0.023)	(0.057)	(0.057)	(0.046)	(0.046)	(0.144)	(0.144)
COVID-19 X Year	0.023	0.092***	-0.005	0.067**	0.108	0.169*	0.129**	0.179**	0.067	0.149
	(0.027)	(0.033)	(0.027)	(0.031)	(0.071)	(0.094)	(0.061)	(0.073)	(0.177)	(0.243)
Cigarettes price		-0.404***		-0.420***		-0.355		-0.292		-0.482
		(0.132)		(0.149)		(0.285)		(0.315)		(0.578)
Constant	0.565***	2.063***	0.514***	2.070***	0.725***	2.039*	0.637***	1.719	0.904***	2.688
	(0.018)	(0.483)	(0.018)	(0.543)	(0.052)	(1.048)	(0.042)	(1.173)	(0.133)	(2.102)
Baseline 2019	0.7936	0.7936	0.7304	0.7304	0.9905	0.9905	0.8876	0.8876	1.1988	1.1988
N	420368	420368	318136	318136	102232	102232	68432	68432	33800	33800
Degrees of freedom	4041	4041	3058	3058	982	982	657	657	324	324
Clusters (individuals)	4042	4042	3059	3059	983	983	658	658	325	325

* p<0.1, ** p<0.05, *** p<0.01

Supplementary Table S5. Full Regression Table on Cigarette Consumption.

a

	Cigarettes purchase rate									
	**All**	**All+price**	**Non-smokers**	**Non-smokers+price**	**Smokers**	**Smokers+price**	**Occasionals**	**Occasionals+price**	**Regulars**	**Regulars+price**
Spring	0.002	0.001	-0.000	-0.000	0.009*	0.005	0.008*	0.007	0.013	0.000
	(0.001)	(0.001)	(0.000)	(0.000)	(0.005)	(0.006)	(0.004)	(0.005)	(0.014)	(0.014)
Summer	-0.001	-0.000	-0.000	-0.000	-0.003	-0.001	0.006	0.007	-0.023*	-0.017
	(0.001)	(0.001)	(0.000)	(0.000)	(0.005)	(0.005)	(0.005)	(0.005)	(0.014)	(0.014)
Fall	-0.004***	-0.003*	-0.000	-0.000	-0.014***	-0.010*	0.001	0.002	-0.044***	-0.032**
	(0.001)	(0.001)	(0.000)	(0.000)	(0.005)	(0.005)	(0.005)	(0.005)	(0.013)	(0.014)
COVID-19	0.006***	0.006***	0.000	0.000	0.026***	0.025***	0.000	-0.000	0.077***	0.074***
	(0.002)	(0.002)	(0.000)	(0.000)	(0.007)	(0.007)	(0.006)	(0.005)	(0.018)	(0.018)
Year (2020)	0.003	0.003	0.003***	0.003***	0.003	0.003	0.003	0.003	0.004	0.004
	(0.002)	(0.002)	(0.000)	(0.000)	(0.008)	(0.008)	(0.006)	(0.006)	(0.022)	(0.022)
COVID-19 X Year	-0.016***	-0.012***	0.001	0.001	-0.066***	-0.053***	-0.008	-0.005	-0.183***	-0.149***
	(0.002)	(0.002)	(0.000)	(0.001)	(0.009)	(0.008)	(0.006)	(0.006)	(0.023)	(0.021)
Cigarettes price		-0.019***		0.000		-0.077***		-0.016		-0.200***
		(0.006)		(0.002)		(0.023)		(0.018)		(0.057)
Constant	0.045***	0.113***	0.000	-0.001	0.183***	0.469***	0.054***	0.114*	0.446***	1.187***
	(0.001)	(0.021)	(0.000)	(0.007)	(0.006)	(0.082)	(0.004)	(0.066)	(0.015)	(0.208)
Baseline 2019	0.0491	0.0491	0	0	0.2021	0.2021	0.0576	0.0576	0.4946	0.4946
N	420368	420368	318136	318136	102232	102232	68432	68432	33800	33800
Degrees of freedom	4041	4041	3058	3058	982	982	657	657	324	324
Clusters (individuals)	4042	4042	3059	3059	983	983	658	658	325	325

* p<0.1, ** p<0.05, *** p<0.01

b

	Cigarettes quantity									
	**All**	**All+price**	**Non-smokers**	**Non-smokers+price**	**Smokers**	**Smokers+price**	**Occasionals**	**Occasionals+price**	**Regulars**	**Regulars+price**
Spring	0.397***	0.193	0.002	-0.006	1.624***	0.812	0.333	0.225	4.237***	2.001
	(0.134)	(0.138)	(0.032)	(0.027)	(0.543)	(0.560)	(0.205)	(0.208)	(1.579)	(1.640)
Summer	-0.012	0.084	-0.034	-0.030	0.055	0.439	0.077	0.128	0.012	1.068
	(0.135)	(0.131)	(0.027)	(0.027)	(0.547)	(0.533)	(0.236)	(0.228)	(1.587)	(1.544)
Fall	-0.239*	-0.050	-0.029	-0.021	-0.892*	-0.142	0.010	0.110	-2.720*	-0.652
	(0.132)	(0.129)	(0.026)	(0.027)	(0.537)	(0.524)	(0.240)	(0.220)	(1.547)	(1.522)
COVID-19	0.630***	0.587***	0.018	0.017	2.535***	2.362***	-0.114	-0.137	7.899***	7.423***
	(0.168)	(0.165)	(0.024)	(0.025)	(0.684)	(0.669)	(0.256)	(0.253)	(1.971)	(1.930)
Year (2020)	0.215	0.221	0.097***	0.098***	0.582	0.605	0.176	0.179	1.403	1.467
	(0.175)	(0.176)	(0.019)	(0.019)	(0.719)	(0.721)	(0.253)	(0.253)	(2.114)	(2.122)
COVID-19 X Year	-0.949***	-0.400**	0.059**	0.081**	-4.083***	-1.897**	0.542*	0.834***	-13.447***	-7.424***
	(0.215)	(0.188)	(0.025)	(0.037)	(0.875)	(0.764)	(0.290)	(0.297)	(2.503)	(2.203)
Cigarettes price		-3.213***		-0.131		-12.802***		-1.710		-35.260***
		(0.679)		(0.138)		(2.736)		(1.079)		(7.845)
Constant	2.784***	14.686***	0.000	0.486	11.449***	58.874***	1.663***	7.998**	31.262***	161.879***
	(0.124)	(2.475)	(0.009)	(0.509)	(0.511)	(9.976)	(0.186)	(3.981)	(1.498)	(28.571)
Baseline 2019	3.3376	3.3376	0	0	13.7240	13.7240	1.16816	1.16816	38.1053	38.1053
N	420368	420368	318136	318136	102232	102232	68432	68432	33800	33800
Degrees of freedom	4041	4041	3058	3058	982	982	657	657	324	324
Clusters (individuals)	4042	4042	3059	3059	983	983	658	658	325	325

* p<0.1, ** p<0.05, *** p<0.01

Supplementary Table S6. Regressions on the Impact of COVID-19 during the Strict Lockdown Period and the Post-lockdown Period on Cigarette Purchase Rate (a) and Cigarette Quantity (b).

a

	Cigarette purchase rate during different periods									
	**All**	**All+price**	**Non-smokers**	**Non-smokers+price**	**Smokers**	**Smokers+price**	**Occasionals**	**Occasionals+price**	**Regulars**	**Regulars+price**
Spring	0.001	0.001	-0.000	0.000	0.004	0.004	0.005	0.004	0.002	0.002
	(0.001)	(0.001)	(0.000)	(0.000)	(0.004)	(0.004)	(0.003)	(0.003)	(0.011)	(0.010)
Summer	0.001	-0.001	0.000	0.000	0.004	-0.004	0.005	0.004	0.002	-0.019*
	(0.001)	(0.001)	(0.000)	(0.000)	(0.004)	(0.004)	(0.003)	(0.004)	(0.011)	(0.011)
Fall	-0.001	-0.003***	-0.000	0.000	-0.002	-0.013***	-0.000	-0.002	-0.007	-0.036***
	(0.001)	(0.001)	(0.000)	(0.000)	(0.005)	(0.005)	(0.004)	(0.004)	(0.013)	(0.012)
Cigarettes price	-0.035***	-0.009*	0.003	0.003*	-0.151***	-0.047**	-0.021	-0.004	-0.415***	-0.133**
	(0.006)	(0.005)	(0.002)	(0.002)	(0.024)	(0.021)	(0.017)	(0.017)	(0.060)	(0.054)
Year (2020)	-0.005***	-0.001	0.003***	0.004***	-0.028***	-0.015**	-0.001	0.003	-0.082***	-0.052***
	(0.002)	(0.002)	(0.000)	(0.000)	(0.006)	(0.007)	(0.005)	(0.006)	(0.015)	(0.019)
Lockdown	0.000		0.000		-0.000		-0.004		0.008	
	(0.001)		(0.000)		(0.005)		(0.004)		(0.013)	
Year X Lockdown	-0.000		0.001**		-0.005		0.005		-0.025	
	(0.002)		(0.001)		(0.007)		(0.005)		(0.017)	
Post-lockdown		0.005***		-0.000		0.021***		0.005		0.053***
		(0.001)		(0.000)		(0.006)		(0.004)		(0.015)
Year X Post-lockdown		-0.011***		-0.001		-0.044***		-0.009		-0.115***
		(0.002)		(0.001)		(0.008)		(0.006)		(0.020)
Constant	0.177***	0.080***	-0.010	-0.012*	0.760***	0.366***	0.133**	0.070	2.029***	0.966***
	(0.022)	(0.020)	(0.006)	(0.007)	(0.087)	(0.078)	(0.060)	(0.063)	(0.222)	(0.198)
N	420368	420368	318136	318136	102232	102232	68432	68432	33800	33800
Degrees of freedom	4041	4041	3058	3058	982	982	657	657	324	324
Clusters (individuals)	4042	4042	3059	3059	983	983	658	658	325	325

* p<0.1, ** p<0.05, *** p<0.01

b

Cigarette quantity during different periods

	All	All+price	Non-smokers	Non-smokers+price	Smokers	Smokers+price	Occasionals	Occasionals+price	Regulars	Regulars+price
Spring	0.452***	0.550***	0.011	0.061**	1.821***	2.072***	0.397**	0.480***	4.704***	5.295***
	(0.116)	(0.113)	(0.015)	(0.029)	(0.471)	(0.451)	(0.173)	(0.160)	(1.368)	(1.308)
Summer	0.392***	0.304**	-0.002	0.035	1.619***	1.141**	0.193	0.289	4.507***	2.864*
	(0.112)	(0.128)	(0.014)	(0.029)	(0.455)	(0.520)	(0.147)	(0.199)	(1.330)	(1.517)
Fall	0.249**	0.113	-0.002	0.034	1.030**	0.360	0.118	0.234	2.878**	0.615
	(0.115)	(0.121)	(0.012)	(0.027)	(0.472)	(0.491)	(0.165)	(0.202)	(1.385)	(1.428)
Cigarettes price	-2.923***	-1.249**	0.156*	0.217**	-12.505***	-5.813**	0.287	-0.393	-38.403***	-16.786**
	(0.627)	(0.569)	(0.089)	(0.100)	(2.540)	(2.316)	(0.935)	(1.036)	(7.244)	(6.649)
Year (2020)	-0.250*	0.268	0.098***	0.165***	-1.334**	0.589	0.432*	0.694**	-4.910***	0.377
	(0.139)	(0.178)	(0.017)	(0.031)	(0.570)	(0.726)	(0.226)	(0.287)	(1.646)	(2.120)
Lockdown	-0.178		-0.008		-0.707		-0.269		-1.594	
	(0.115)		(0.009)		(0.471)		(0.168)		(1.384)	
Year X Lockdown	0.622***		0.144**		2.111***		0.816***		4.734**	
	(0.184)		(0.056)		(0.735)		(0.314)		(2.124)	
Post-lockdown		0.427***		-0.014		1.798***		-0.031		5.503***
		(0.135)		(0.015)		(0.550)		(0.164)		(1.611)
Year X Post-lockdown		-1.015***		-0.079**		-3.928***		-0.039		-11.801***
		(0.217)		(0.036)		(0.880)		(0.303)		(2.537)
Constant	13.898***	7.435***	-0.578*	-0.828**	58.943***	33.150***	0.482	2.895	177.305***	94.404***
	(2.316)	(2.097)	(0.330)	(0.374)	(9.380)	(8.524)	(3.494)	(3.850)	(26.699)	(24.450)
N	420368	420368	318136	318136	102232	102232	68432	68432	33800	33800
Degrees of freedom	4041	4041	3058	3058	982	982	657	657	324	324
Clusters (individuals)	4042	4042	3059	3059	983	983	658	658	325	325

* p<0.1, ** p<0.05, *** p<0.01

Note: the variable *Lockdown weeks* refers to the period covering week 11-19 of each year, while the variable *post-lockdown weeks* indicates the period of time from week 12 to week 52 of each year. The two variables are also interacted with the *Year* dummy in the model.

Supplementary Table S7. Regressions on the Impact of COVID-19 on Cigarette Quantity Before the Price Increase.

	Cigarettes quantity week 11 to week 27									
	**All**	**All+price**	**Non-smokers**	**Non-smokers+price**	**Smokers**	**Smokers+price**	**Occasionals**	**Occasionals+price**	**Regulars**	**Regulars+price**
Spring	0.126	0.131	0.018	0.019	0.463	0.479	-0.045	-0.045	1.491	1.538
	(0.146)	(0.146)	(0.023)	(0.023)	(0.596)	(0.597)	(0.232)	(0.231)	(1.743)	(1.743)
Summer	0.018	0.067	-0.010	-0.005	0.106	0.292	-0.281	-0.278	0.888	1.444
	(0.163)	(0.164)	(0.018)	(0.019)	(0.666)	(0.671)	(0.256)	(0.253)	(1.949)	(1.964)
Fall	-0.163	-0.108	0.004	0.010	-0.682	-0.478	-0.175	-0.172	-1.707	-1.096
	(0.170)	(0.172)	(0.019)	(0.020)	(0.697)	(0.703)	(0.256)	(0.255)	(2.043)	(2.063)
COVID-19	0.688***	0.629***	-0.004	-0.011	2.841***	2.620***	0.180	0.176	8.230***	7.567***
	(0.158)	(0.157)	(0.019)	(0.020)	(0.641)	(0.637)	(0.281)	(0.279)	(1.818)	(1.814)
Year (2020)	0.215	0.219	0.097***	0.098***	0.582	0.598	0.176	0.177	1.403	1.450
	(0.175)	(0.176)	(0.019)	(0.019)	(0.719)	(0.720)	(0.253)	(0.253)	(2.115)	(2.117)
COVID-19 X Year	-0.522***	-0.360*	0.072**	0.090**	-2.373***	-1.764**	0.710**	0.720**	-8.616***	-6.791***
	(0.183)	(0.190)	(0.034)	(0.039)	(0.744)	(0.773)	(0.294)	(0.306)	(2.131)	(2.230)
Cigarettes price		-4.480***		-0.503		-16.856***		-0.260		-50.457***
		(1.295)		(0.332)		(5.204)		(2.163)		(14.962)
Constant	2.784***	-3.087*	-0.000	-0.659	11.449***	-10.644	1.663***	1.322	31.262***	-34.871*
	(0.124)	(1.730)	(0.008)	(0.440)	(0.505)	(6.953)	(0.163)	(2.862)	(1.477)	(19.982)
N	319318	319318	241661	241661	77657	77657	51982	51982	25675	25675
Degrees of freedom	4041	4041	3058	3058	982	982	657	657	324	324
Clusters (individuals)	4042	4042	3059	3059	983	983	658	658	325	325

* p<0.1, ** p<0.05, *** p<0.01

Supplementary Table S8. Regression Table on Money Spent in Cigarettes in US$

	Money spent on cigarettes				
	All	Non-smokers	Smokers	Occasionals	Regulars
Spring	0.067	-0.008	0.299	0.084	0.734
	(0.045)	(0.012)	(0.182)	(0.072)	(0.530)
Summer	0.013	-0.016	0.103	0.069	0.171
	(0.048)	(0.011)	(0.194)	(0.082)	(0.563)
Fall	-0.020	-0.009	-0.054	0.081	-0.327
	(0.048)	(0.011)	(0.196)	(0.092)	(0.563)
COVID-19	0.207***	0.010	0.818***	-0.068	2.610***
	(0.057)	(0.010)	(0.231)	(0.087)	(0.667)
Year (2020)	0.084	0.030***	0.250	0.070	0.616
	(0.056)	(0.006)	(0.230)	(0.082)	(0.677)
COVID-19 X Year	-0.184**	0.026***	-0.838***	0.304***	-3.149***
	(0.073)	(0.009)	(0.296)	(0.105)	(0.858)
Constant	0.896***	0.000	3.683***	0.528***	10.071***
	(0.041)	(0.003)	(0.169)	(0.064)	(0.491)
Baseline	1.12	0	4.61	0.53	12.87
N	420368	318136	102232	68432	33800
Degrees of freedom	4041	3058	982	657	324
Clusters (individuals)	4042	3059	983	658	325

* p<0.1, ** p<0.05, *** p<0.01

Note: conversion rate 1 US$ to 6.26 DKK as of Aug 4, 2021 (Google Finance).

Supplementary Table S9. Danish Media Coverage on the Potential Correlation between Smoking and the Risk of getting COVID-19.

Source	Description	Date	Headline	Link	Correlation between smoking and risk of getting COVID-19
TV2	Danish broadcasting corporation	March 23rd 2020	*Smokers at increased risk of dying from corona*	here	+
Dagens Medecin	Specialized medical newspaper	March 27th, 2020	*Smokers at increased risk of becoming infected with coronavirus.*	here	+
Lunge	Lung patient association	March 27th, 2020	*Smokers at increased risk of becoming infected with coronavirus.*	here	+
DR	Danish broadcasting corporation	April 3rd, 2020	*Read the answers about smoking and corona: There is a huge gain already after eight hours of stopping.*	here	+
Politiken	Major newspaper	April 6th, 2020	*Stopping smoking makes you better at handling COVID-19 infection*	here	+
Newsbreak	News agency	April 6th, 2020	*Higher risk of COVID-19 for smokers*	here	+
Berlingske	Major newspaper	April 23rd, 2020	*Spectacular studies: Smokers may be at lower risk of getting coronavirus*	here	Perhaps -
Danish Ministry of Health	Agency for prevention against infectious diseases and biological threats	May 25th, 2020	*Subjects with higher risk of COVID-19*	here	Not clear
Kristligt Dagblad	Major newspaper	May 30th 2020	*Corona and smoking experience in Norway*	here	+/-
Berlingske	Major newspaper	July 1st, 2020	*Experience from COVID-19 can save lives*	here	+
Fyens Stiftstidende	Major newspaper	September 9th 2020	*Smoking is much more deadly for COVID-19*	here	+
Danish Ministry of Health	Agency for prevention against infectious diseases and biological threats	March 12th, 2021	*Subjects with higher risk of COVID-19*	here	+

Note: the overview does not seek to be exhaustive and does not include Danish regional newspapers or local television programs, for instance.
