# Supplementary material for: Sustained decline in tobacco purchasing in Denmark during the COVID-19 pandemic
Source: Commun Med (Lond). 2022 Aug 2;2:96. doi: 10.1038/s43856-022-00160-1 (PMC9344799; doi:10.1038/s43856-022-00160-1)
Supplement: Supplementary file 3 — Reporting Summary [file 43856_2022_160_MOESM3_ESM.pdf]

nature research

Corresponding author(s): Sally Sadoff

Last updated by author(s): May 26, 2022

Reporting Summary

Nature Research wishes to improve the reproducibility of the work that we publish. This form provides structure for consistency and transparency in reporting. For further information on Nature Research policies, see our Editorial Policies and the Editorial Policy Checklist.

Statistics

For all statistical analyses, confirm that the following items are present in the figure legend, table legend, main text, or Methods section.

n/a	Confirmed	
☐	☒	The exact sample size (*n*) for each experimental group/condition, given as a discrete number and unit of measurement
☐	☒	A statement on whether measurements were taken from distinct samples or whether the same sample was measured repeatedly
☐	☒	The statistical test(s) used AND whether they are one- or two-sided<br>*Only common tests should be described solely by name; describe more complex techniques in the Methods section.*
☐	☒	A description of all covariates tested
☒	☐	A description of any assumptions or corrections, such as tests of normality and adjustment for multiple comparisons
☒	☐	A full description of the statistical parameters including central tendency (e.g. means) or other basic estimates (e.g. regression coefficient) AND variation (e.g. standard deviation) or associated estimates of uncertainty (e.g. confidence intervals)
☒	☐	For null hypothesis testing, the test statistic (e.g. *F*, *t*, *r*) with confidence intervals, effect sizes, degrees of freedom and *P* value noted<br>*Give P values as exact values whenever suitable.*
☒	☐	For Bayesian analysis, information on the choice of priors and Markov chain Monte Carlo settings
☒	☐	For hierarchical and complex designs, identification of the appropriate level for tests and full reporting of outcomes
☒	☐	Estimates of effect sizes (e.g. Cohen's *d*, Pearson's *r*), indicating how they were calculated

Our web collection on statistics for biologists contains articles on many of the points above.

Software and code

Policy information about availability of computer code

Data collection	Data was purchased from a partner company who had previously collected the data as part of their core business.
Data analysis	The data was cleaned, managed and analyzed via the data software Stata 17.

For manuscripts utilizing custom algorithms or software that are central to the research but not yet described in published literature, software must be made available to editors and reviewers. We strongly encourage code deposition in a community repository (e.g. GitHub). See the Nature Research guidelines for submitting code & software for further information.

Data

Policy information about availability of data

All manuscripts must include a data availability statement. This statement should provide the following information, where applicable:

- Accession codes, unique identifiers, or web links for publicly available datasets
- A list of figures that have associated raw data
- A description of any restrictions on data availability

The data that support the findings of this study is are not publicly available as dictated by contractual agreement between the University of Copenhagen and the data collecting partner company. Data are however available from the authors upon reasonable request and with permission of the partner company, but restrictions apply to the availability of these data. The raw data associated with the figures is not publicly available as dictated by contractual agreement between the University of Copenhagen and the data collecting partner company.

Field-specific reporting

Please select the one below that is the best fit for your research. If you are not sure, read the appropriate sections before making your selection.

☐ Life sciences ☒ Behavioural & social sciences ☐ Ecological, evolutionary & environmental sciences

For a reference copy of the document with all sections, see nature.com/documents/nr-reporting-summary-flat.pdf

Behavioural & social sciences study design

All studies must disclose on these points even when the disclosure is negative.

Study description	Quantitative research study on the impact of COVID-19 on smoking purchases from a within-subject analysis of a longitudinal data set of purchasing behavior from 2019-2020 among a national sample of the Danish population (n=4042).
Research sample	National sample of the Danish population (n=4042). A subsample of our participants self-reported demographic characteristics via the app during the sign-up process: gender (68% report), age (68.5% report), employment status (70.4% report), household type (71.5% report). Compared to the overall population, our participants are on average more likely to be female, to cover younger age groups, to have a higher income, to have children in the household and to be more heavily drawn from the capital region (Copenhagen). Representativeness issues are addressed in the analysis and no impact on the results is found.
Sampling strategy	There was no sampling strategy for this study, as individuals were not aware that their cigarette purchase was being observed, and no compensation was involved. Users would typically find out about these apps through marketing campaigns run by the developer company.
Data collection	Data was collected via smartphone apps. Users of the apps created a profile and connected it with their supermarket membership (which was also connected with a popular electronic receipt system). This setting allowed the app the collection of supermarket purchase data. The data was collected in order to provide the core service of the commercial apps: providing an overview of the items purchased at the supermarket together with other kinds of informations (carbon footprint and monetary expenses, respectively).
Timing	Data collection started on the date that users downloaded the app and connected it with their electronic receipt system and supermarket membership. Historic data on their supermarket purchases were also available. Purchase data at supermarkets were observed for the year 2019 and 2020.
Data exclusions	The original sample included 5092 app users. Only people with continuous and consistent data for both periods (2019 and 2020) were kept in the dataset (N=4042).
Non-participation	Participants were not aware of their participation since the dataset was purchased from a company partner.
Randomization	Participants were not allocated in experimental group. The study is based on a within-subject analysis.

Reporting for specific materials, systems and methods

We require information from authors about some types of materials, experimental systems and methods used in many studies. Here, indicate whether each material, system or method listed is relevant to your study. If you are not sure if a list item applies to your research, read the appropriate section before selecting a response.

Materials & experimental systems

n/a	Involved in the study	
☒	☐	Antibodies
☒	☐	Eukaryotic cell lines
☒	☐	Palaeontology and archaeology
☒	☐	Animals and other organisms
☐	☒	Human research participants
☒	☐	Clinical data
☒	☐	Dual use research of concern

Methods

n/a	Involved in the study	
☒	☐	ChIP-seq
☒	☐	Flow cytometry
☒	☐	MRI-based neuroimaging

Human research participants

Policy information about studies involving human research participants

Population characteristics	See above.
Recruitment	There was no recruitment for this study, as individuals were not aware that their cigarette purchase was being observed, and

Recruitment	no compensation was involved. Users would typically find out about these apps through marketing campaigns run by the developer company.
Ethics oversight	*Identify the organization(s) that approved the study protocol.*

Note that full information on the approval of the study protocol must also be provided in the manuscript.
